# High-resolution geoelectrical characterization and monitoring of natural fluids emission systems to understand possible gas leakages from geological carbon storage reservoirs

**DOI:** 10.1038/s41598-023-45637-8

**Published:** 2023-10-30

**Authors:** Rosanna Salone, Claudio De Paola, Rolando Carbonari, Francesco Rufino, Rosario Avino, Stefano Caliro, Emilio Cuoco, Alessandro Santi, Rosa Di Maio

**Affiliations:** 1 Dipartimento di Scienze della Terra, dell’Ambiente e delle Risorse, Università di Napoli Federico II, 80126 Naples, Italy; 2 Institute of Earth Sciences, The Hebrew University of Jerusalem, 9190401 Jerusalem, Israel; 3 Istituto Nazionale di Geofisica e Vulcanologia, Osservatorio Vesuviano, 80124 Naples, Italy

**Keywords:** Geophysics, Geochemistry

## Abstract

Gas leakage from deep geologic storage formations to the Earth’s surface is one of the main hazards in geological carbon sequestration and storage. Permeable sediment covers together with natural pathways, such as faults and/or fracture systems, are the main factors controlling surface leakages. Therefore, the characterization of natural systems, where large amounts of natural gases are released, can be helpful for understanding the effects of potential gas leaks from carbon dioxide storage systems. In this framework, we propose a combined use of high-resolution geoelectrical investigations (i.e. resistivity tomography and self-potential surveys) for reconstructing shallow buried fracture networks in the caprock and detecting preferential gas migration pathways before it enters the atmosphere. Such methodologies appear to be among the most suitable for the research purposes because of the strong dependence of the electrical properties of water-bearing permeable rock, or unconsolidated materials, on many factors relevant to CO_2_ storage (i.e. porosity, fracturing, water saturation, etc.). The effectiveness of the suggested geoelectrical approach is tested in an area of natural gas degassing (mainly CH_4_) located in the active fault zone of the Bolle della Malvizza (Southern Apennines**,** Italy), which could represent a natural analogue of gas storage sites due to the significant thicknesses (hundreds of meters) of impermeable rock (caprock) that is generally required to prevent carbon dioxide stored at depth from rising to the surface. The obtained 3D geophysical model, validated by the good correlation with geochemical data acquired in the study area and the available geological information, provided a structural and physical characterization of the investigated subsurface volume. Moreover, the time variations of the observed geophysical parameters allowed the identification of possible migration pathways of fluids to the surface.

## Introduction

High-resolution 3D electrical resistivity tomography (ERT) coupled with self-potential (SP) surveys are geophysical prospecting methods that have been used for several environmental engineering purposes to detect variations in soil conductivity due to fluids of different nature, such gas and/or groundwater. For example, saline waters from landfill leachate^1^, mining waste monitoring^2^, water seepage in dams and related basins^3,4^. In addition, electrical geophysical prospecting has proven to be very useful in recognizing preferential pathways for gas migration to the surface and thus in monitoring carbon dioxide upwelling phenomena^5–7^ as well as methane migration in groundwater^8^. Indeed, it’s well known that methods based on electrical imaging techniques are well suited for site characterization and monitoring of fluid movement and subsurface processes, as the electrical properties of a geological setting are often dominated by the electrical properties of the fluids within it. Therefore, these techniques are particularly sensitive to changes in both presence and nature of formation fluids. In the light of recent investigations, such geophysical methods are become relevant for the detection and monitoring of the most favorable geological environments for anthropogenic CO_2_ storage [Ref.^9^ and references therein]. Overall, these methods represent state-of-the-art techniques that have been used in recent years to reduce atmospheric greenhouse gas emissions^10–12^.

The present study proposes the integration of high-resolution 3D electrical resistivity tomography (ERT) and self-potential (SP) surveys to identify possible shallow fault/fracture systems, which could represent preferential pathways for fluids migration from deep geological storage formations to the surface. The need to integrate these two investigation methods is based on (i) the nowadays-recognized dependence of the electrical resistivity of rocks and soils on many geochemical factors, such as water salinity, presence of gas phases, cation exchange capacity of minerals, and temperature^13–15^, and (ii) the ability of the SP signal to highlight deep anomalous natural electric charge polarizations (streaming potential) associated with the underground fluid circulation. The combined use of ERT and SP should therefore provide a more effective assessment of the fluid distribution within the investigated subsurface volume. The repetition of the integrated prospecting over time is also suggested as a powerful tool for time-lapse monitoring of possible gas leakages from the reservoir in terms of potential time variations of the observed electrical parameters.

The proposed approach is tested in the active fault zone of the Bolle della Malvizza (Southern Apennines, Italy), which is affected by the ascent of fluids to the surface, as evidenced by the presence of mud volcanoes (MVs). The test site was selected on the basis of its geological setting, which consists of a thick clay formation (several hundred meters) overlying a carbonate basement. This clay formation could well represent a natural analogue of the caprock present in gas storage sites. Here, the emitted fluids, saline waters and gas phase, consisting mainly of methane (CH_4_), are embedded in synorogenic Tertiary formations^16^.

To further strengthen and validate the proposed integrated high-resolution ERT and SP techniques, a geochemical survey, consisting of soil gas flux measurements, water and gas sampling for chemical and isotopic analyses, has been carried out in the geophysical study area.

## Geological and tectonic background of Bolle della Malvizza area (Avellino, Southern Italy)

**Figure 1:**
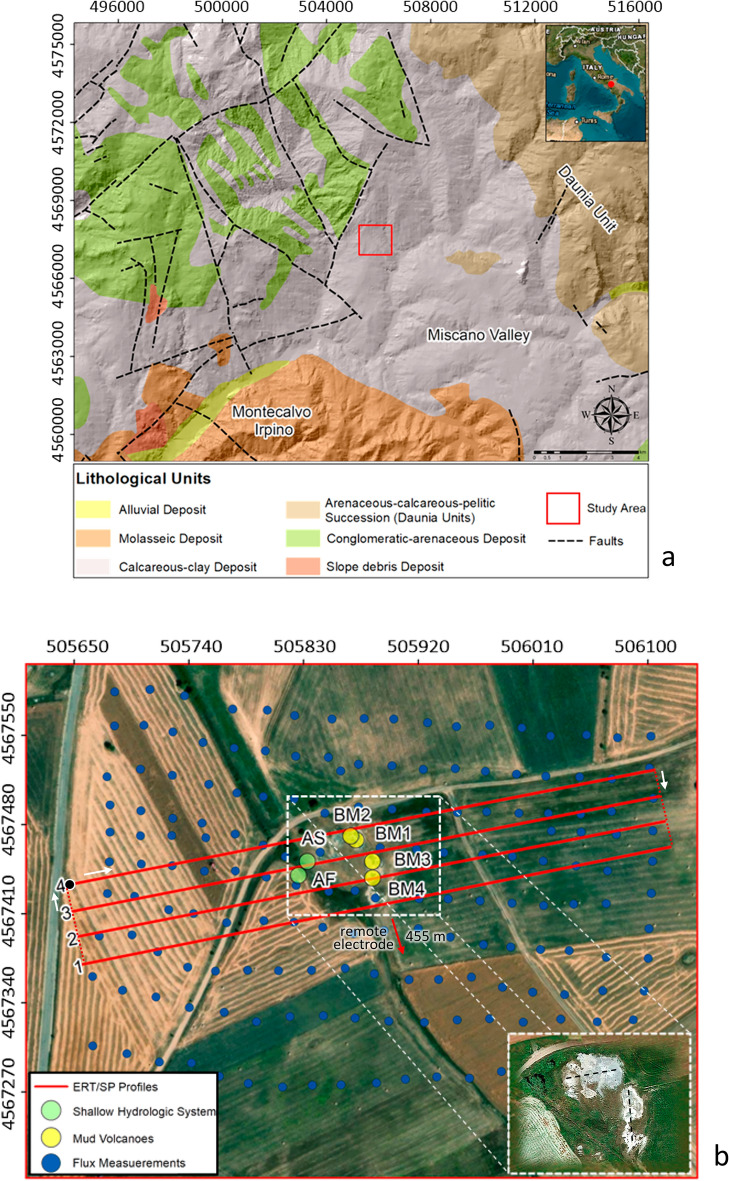
(**a**) Simplified geological scheme of the survey area from Italian National Institute for Environmental Protection and Research (modified from Ref.^17,18^); (**b**) Geographical setting of the Bolle della Malvizza survey area (Avellino, Southern Italy). The four continuous red lines show the location of the ERT profiles; the continuous and dashed red lines form the closed circuit along which the SP survey was carried out, where the white arrows and the black dot indicate the progression of the SP measurements and the final reference point of the whole SP data set [see Ref.^29^], respectively; the blue dots indicate the flux measurement points; the inset shows the orientation of some mud volcanoes (MVs) (image from Google Earth™). The figure was created using the ArcMap 10.2.2 software (https://www.esri.com/en-us/arcgis/products/arcgis-desktop/resources) and postprocessed through the Surfer 16 software (https://www.goldensoftware.com/products/surfer/).

The area of the Bolle della Malvizza (Fig. 1a) is a small complex of mud volcanoes located in the Apennines mountains chain in Campania region (Southern Italy). The study area lies on a plateau in the Miscano valley (Fig. 1a) at an elevation of 518 m above sea level (a.s.l.). In accordance with the illustrative notes of the geological map of Italy^17,18^, the main lithological unit outcropping in the Bolle della Malvizza area consists of clays and marls of Miocene age intercalated with interlayers and/or complex layers of limestones and marly limestones. This formation is defined in hydrogeological terms by Bruno et al.^19^ as a “*clay-marly”* complex. The prevailing type and grade of permeability of this formation are by porosity and very low, respectively. The complex is considered impermeable and plays the hydrogeological role of an aquiclude with respect to the more permeable Daunia formation^20^, consisting of an arenaceous-calcareous-pelitic succession. The latter is the most important deposit outcropping in the peripheral areas, where molassic and/or conglomeratic arenaceous material is subordinately found (Fig. 1a). Specifically, the study area is located in the so-called Irpino-Daunia area, in a transition zone between the Molisano-Sannitic (Upper Pliocene) and Campano-Lucano (Lower Pleistocene) segments of the outer sector of the southern Apennine chain. The two segments intersect the Ofanto synform to the north, where the WNW-ESE compressional structures of the Campano-Lucano sector chain cross the NNW-SSE and NW–SE structures of the Molisano-Sannitic sector^21,22^. In terms of sedimentology and stratigraphy, the Bolle della Malvizza area is characterized by the presence of the Meso-Cenozoic sedimentary sequence of the Lagonegro-Molise basin^23^, consisting of the Fortore Unit and the Vallone del Toro Unit, separated by the regional high-angle Benevento-Buonalbergo normal fault^24^. In particular, the Fortore Unit is formed by a 2000 m thick succession of clays, varicolored argillites and marls with intercalations of calcilutites and calcarenites, locally with jaspers (AVF, Varicolored Clays of Fortore Unit). Subsequently, the succession evolves to volcanoclastic deposits, numidic sandstones, post-numidic deposits and turbiditic silicoclastic sandstones^25^. On the other hand, the Vallone del Toro Unit consists of a 2000 m thick Upper Oligocene-Middle Burdigalian succession, with alternating calcarenites, argillites and calcareous breccias at the base, and Upper Burdigalian-Lower Messinian calcarenites and Upper Messinian-Lower Zanclean varicolored clays with gypsum and sulfur layers at the top^25^. However, a 2180 m deep well, located about 1.5 km away from the Bolle della Malvizza (i.e. the Casalbore 001 Well)^26^, suggests that the area targeted by the geophysical prospection is exclusively composed of the AVF succession (Fortore Unit). More in detail, the investigated area is characterized by the presence of mud volcanoes related to the Neogene Apennine tectonics, which generated a network of dislocations, highly permeable to fluids migration^27^. Malvizza mud volcanoes and the discharged fluids appear to be related to phenomena resulting from intense deep alteration associated with fluid-rock interactions that commonly occur along active and permeable faults^28^. Indeed, the area is characterized by numerous fault segments with SW-NE, NW–SE and N-S trends associated with local tectonic activity, where MVs took place (Fig. 1b). They seem to be oriented along soil discontinuities (i.e. faults or fractures) that provide preferential pathways for ascending gas to the surface.

Previous geochemical studies of water and gas samples collected in the Bolle della Malvizza area [Ref.^16^ and references therein] revealed marked Na-Cl composition in the water samples and high CH_4_ content in the gas samples. The authors suggest that the high salinity of the water is probably due to hydrocarbon-associated brines, as supported by the high concentrations of CH_4_ in the gas phase. In particular, they hypothesize that the Na-Cl component derives from fossil waters trapped either in Miocene synorogenic sediments or in Plio-Quaternary sediments and volcanics. Therefore, from a physical point of view, the thick clay cover is expected to be highly conductive due to the interaction of the ascending saline water with the clay minerals, which are characterized by a strong ion-exchange capacity. This in turn would account for a greater amount of dissolved ions, which could lead to significant natural electric charge accumulations at the interfaces between media with different electrical properties. These features guided the selection of this area as an analogue gas storage site. Indeed, our hypothesis is that high-resolution geoelectrical surveys are capable of highlighting even small variations in the distribution of the electrical parameters within highly conductive clay formations, which are likely to be associated with possible fluid migration along permeable fracture systems and/or faults; the latter representing preferential pathways for fluid/gas ascending to the Earth’s surface. Specifically, for the selected test site, the fluid migration, essentially highly saline water, is expected to be driven by the rise of natural methane gas derived from an oil reservoir in organic-rich Tertiary sediments^16^.

The coupled geoelectrical techniques can also be applied for monitoring purposes in the context of gas sequestration and storage. However, the rising fluids would essentially consist of water mixed with CO_2_, the latter coming from possible leakage from deep geological reservoirs.

## Materials and methods

### Geoelectrical measurements

Integrated high-resolution ERT and SP geoelectrical surveys were performed at the Bolle della Malvizza test site (Avellino, Southern Italy, see Fig. 1b). Both surveys were carried out in two different periods, namely March (winter survey) and July (summer survey), in order to detect and interpret variations in the observed geophysical parameters likely related to differences in the fluid/gas migration along possible fracture systems.

#### ERT survey and data processing

The ERT survey was performed by applying the so-called pseudo-3D ERT imaging technique^30,31^, which consists in placing the electrodes along parallel profiles to realize 2D apparent resistivity sections to be inverted with 3D inversion algorithms^32,33^. For the study area, the pseudo-3D ERT survey was carried out along the four parallel profiles shown in Fig. 1b with red lines, spaced 20 m apart. The data were acquired using the IRIS Syscal Pro Switch georesistivimeter (IRIS Instruments) equipped with multi-electrode cables consisting of 48 electrodes spaced 10 m apart and using the pole-dipole array, which allows faster data acquisition times and greater investigation depths than other electrode configurations, as well as a good horizontal resolution^34^. According to Yadav et al.^35^, the general pole-dipole configuration was adopted. Specifically, the remote current electrode, which is theoretically placed at infinity, was fixed at 455 m from profile 1 perpendicular to the centre of the potential dipole located approximately in the central part of the profile (red arrow in Fig. 1b). In particular, each 2D acquisition was made with a measurement sequence consisting of 198 current injections and 1337 measurement quadrupoles reaching an investigation depth of about 90 m below ground level (b.g.l.). The RES3DINV software^36^ was used to invert the acquired apparent resistivity data, taking into account the profiles topography ranging from about 562 m to 571 m a.s.l. Finite element and complete Gauss–Newton methods were adopted to relate the model parameters to the 3D model response and to control the change in the model parameters, respectively.

#### SP survey and data processing

The SP measurements were performed continuously along the four ERT profiles in Fig. 1b, forming closed circuits, i.e. profiles connected at both ends to other closed profiles (see Fig. 1b), to generate a fully interconnected network^29^. This procedure allows the reduction of cumulative errors, for example due to cultural noise, by applying a closure correction along the circuits (for details about the adopted approach the reader is referred to^29^). Data were collected using a 10 m long receiving dipole consisting of two non-polarizable electrodes made of copper rods immersed in a copper sulfate solution. The SP drops along the measurement network were detected sequentially between each two successive electrode positions once signal stability conditions were reached. A high-impedance voltmeter with a sensitivity of 0.1 mV was used for the data acquisition. To get information on the subsurface distribution of the natural electric charges responsible for the observed surface anomalies, the acquired SP data were interpreted using a 3D tomographic inversion method that also takes into account the topographic effects^37–39^. The method aims to define the underground SP causative sources by evaluating a cross-correlation function between the observed electric field and the field expected from a synthetic electric source located at any point of the investigated subsurface volume. By normalizing the cross-correlation function using the Schwarz inequality, the following charge occurrence probability (COP) function, *η*, is obtained^39^:

1 $$\eta \left({x}_{q},{y}_{q},{z}_{q}\right)={C}_{s}{\int }_{-\infty }^{+\infty }{\int }_{-\infty }^{+\infty }{E}_{s}[x,y,z\left(x,y\right)]\cdot {\mathfrak{I}}_{s}\left[x-{x}_{q}, y-{y}_{q}, z\left(x,y\right)-{z}_{q}\right]dxdy,$$

where *E*_*s*_ and ℑ_*s*_ are, respectively, the natural electric field measured at the surface point P[x,y,z(x,y)] and the synthetic electric field generated by an elementary positive charge located at the generic point of the subsoil with coordinates (xq, yq, zq), while *C*_*s*_ is the normalization factor that limits the variations of the *η* values in the range [− 1, 1]. In synthesis, the distribution of the *η* values obtained for all the points in the explored volume actually provides a tomographic representation of the in-depth polarization source centres, where the maximum positive and negative values indicate the highest probability for the occurrence of positive and negative charge accumulation zones, respectively.

### Geochemical measurements

The geochemical investigation at the Bolle della Malvizza site, focusing on (i) water and (ii) free gases sampling and (iii) soil diffuse degassing measurements, was carried out in the winter season. All measurements and sampling were performed close to the mud volcanoes area, except for three wells (#PB1, #PB2, #PB3 not shown in Fig. 1b; coordinates in Table 1) that were sampled in peripheral areas.

**Table 1:** Chemical analysis of sampled fluids.

Water analyses																
ID	UTM WGS84 33T E	UTM WGS84 33T N	T (°C)	pH	ORP mV	EC mS/cm	HCO_3_ mg/L	F mg/L	Cl mg/L	SO_4_ mg/L	NO_3_ mg/L	Br mg/L	Na mg/L	K mg/L	Mg mg/L	Ca mg/L
BM1	505,871	4,567,468	13.8	8.37	13	18.25	2832.6	8.8	4957.1	5.4	nd	27.9	4159.9	28.5	14.0	8.4
BM2	505,867	4,567,471	12.5	7.99	79	18.32	2617.9	12.5	4892.3	1.6	nd	27.3	3994.1	7.4	21.5	14.8
BM3	505,884	4,567,451	13.8	7.85	− 177	18.53	2780.3	12.6	4880.5	0.9	nd	27.5	4091.3	8.4	16.1	8.4
BM4	505,884	4,567,438	11.0	7.90	− 61	18.20	2784.5	12.4	4860.6	1.0	nd	27.1	4071.6	23.4	16.4	10.0
PB1	505,657	4,567,144	11.5	7.59	161	1.570	477.18	3.5	265.7	112.1	10.1	nd	296.9	3.9	21.3	55.8
PB2	505,395	4,566,796	14.0	7.23	150	1.020	387.48	1.2	93.5	80.5	47.9	nd	86.3	6.4	20.2	123.4
PB3	505,744	4,567,955	12.0	7.35	152	0.634	408.83	0.6	16.6	31.8	0.2	0.1	35.0	6.6	11.3	107.5
AS	505,833	4,567,451	11.1	8.19	130	2.12	574.2	1.0	446.7	120.8	0.1	nd	454.1	14.7	16.6	60.7
AF	505,826	4,567,440	12.1	7.91	143	0.616	266.65	1.0	30.8	35.6	0.2	nd	48.0	6.7	7.9	56.8

Free gases analyses																
ID	UTM WGS84 33T E	UTM WGS84 33T N	% CO_2_	% Ar	% O_2_	% N_2_	% CH_4_	% He								
BM1	505,871	4,567,468	1.19	0.06	0.71	4.26	95.05	0.05								
BM2	505,867	4,567,471	1.13	0.45	9.95	37.74	51.76	0.03								
BM3	505,884	4,567,451	1.34	0.03	0.12	2.23	95.86	0.05								

Dissolved gases analyses																
ID	UTM WGS84 33T E	UTM WGS84 33T N	CO_2_ mmol/L	Ar mmol/L	O_2_ mmol/L	N_2_ mmol/L	CH_4_ mmol/L	He mmol/L								
BM4	505,884	4,567,438	1.3E + 00	2.4E-03	4.0E-03	1.4E-01	1.5E + 00	8.5E-05								
PB1	505,657	4,567,144	4.6E-01	1.8E-02	1.9E-01	8.2E-01	nd	6.6E-06								
PB2	505,395	4,566,796	6.2E-01	1.7E-02	2.5E-01	7.0E-01	nd	3.6E-06								
PB3	505,744	4,567,955	5.9E-01	2.0E-02	1.2E-01	8.6E-01	nd	3.6E-06								

Free gas phase is expressed as %vol.

#### Soil diffuse gas measurements and data processing

Soil gas fluxes were measured using the accumulation chamber method^40^ by a Thearen portable fluxmeter. The latter consists of: (a) a cylindrical accumulation chamber, about 9.5 cm high with an area of ~ 0.034 m^2^; (b) a Licor-830 non-dispersive infrared (NDIR) detector for CO_2_ measurements (range 0–20,000 ppm); (c) a Sensit Thea-CH_4_ laser sensor for CH_4_ measurements (range 0–20,000 ppm); (d) a CAT 62spro portable device. The gas circulates from the chamber to the detectors and comes back into the chamber via a small-diameter plastic tube connected to a 1 L/min gas pump. The gas mixing inside the chamber is guaranteed by a perforated ring-shaped tube, which re-injects the circulating gas. The soil gas fluxes are computed directly in the field based on the gases accumulation inside the chamber over time (concentration increment: dC_gas_/dt). The increment is computed during the measurement according to the relationship between the gas concentration and the time: flux = cf × dC_gas_/dt^40,41^. The parameter cf is a proportionality factor determined experimentally in the laboratory for both detectors, under controlled flux conditions with a wide range of imposed fluxes (from 10 to 32,000 g/m^2^day for CO_2_ and from 0.18 to 2 g/m^2^day for CH_4_), by calculating the slope of the linear best-fits of the imposed gas fluxes versus the measured dC_gas_/dt. Soil gas flux measurements were performed on a regular grid of 25 × 25 m spaced, for a total of 169 points. Moreover, in order to simultaneously determine the soil CO_2_ flux and its isotopic composition, the instrument was equipped with a T-valve in the gas line. A rubber septum was placed at the beginning of the inlet line, allowing additional gas aliquots to be taken for isotopic analysis. The gas samples were collected with a syringe and then injected into 12 ml pre-evacuated vials. For each of the 54 sampled points, two aliquots of different concentrations were collected according to the method proposed by Ref.^41^. The collected gas samples were analyzed within a few days to avoid carbon isotope fractionation, although previous works show that no carbon isotope fractionation occurs in this type of vials even after several days^42–44^. The samples were analyzed using a continuous flow isotope ratio mass spectrometer (Thermo-Finnigan Delta XP) interfaced with a Gasbench II device equipped with an autosampler (δ^13^C standard error ± 0.1‰).

Methane and CO_2_ maps were built using conditional sequential Gaussian simulations (sGs)^45^. This approach is widely used to map diffusive degassing structures (DDS) in volcanic and non-volcanic areas and is preferred to traditional interpolation algorithms because it allows the spatial variability of the measured attributes to be preserved^46–51^. The GSLIB software^52^ was used for this study. Since this approach requires normally distributed data and, in many cases, experimental data are not normally distributed, the *n-score* executable of the GSLIB software was used to transform the original data into a normal distribution (*n-scores* of data). Variograms of normal scores were then computed for both variables and fitted with a standardized spherical model described by the following equations:

2 $$\gamma ={c}_{0}+c \left[1.5\left(\frac{h}{a}\right)-0.5{\left(\frac{h}{a}\right)}^{3}\right], h<a$$

3 $$\gamma =1, h>a,$$

where* c*_*0*_ is the nugget effect,* c* and *a* are the sill and range, respectively, and *h* is the lag distance. The *sgsim* executable was then used to generate several equiprobable realizations of the spatial distribution of the attributes. Using simple Kriging, the mean and variance of the Gaussian conditional cumulative distribution function can be defined at each location^47,52^. Simple kriging estimates and variances were computed according to the semi-variogram of the normal scores data. Finally, simulated *n-scores* were back-transformed to their original values using a back-transformation operation (*backtr*, i.e. inverse of *n-score*)^47^. For this study, a regular grid of 7272 cells (nx = 101, ny = 72 cells) with a cell size of 4 × 4 m was used. One hundred realizations were produced for each dataset and then post-processed to realize the E-type estimate maps. The estimated values are shown as a pointwise linear average of all the realizations^47,52^. In addition, a graphical-statistical approach^41^ was used to partition the populations.

#### Fluids from mud volcanoes, sampling and analyses

Four water samples were collected directly at the emission points at the top of the mud volcanoes (BM samples), while two samples (AS and AF samples) were collected from a small pool on the ground and a drainage channel in the surrounding area, respectively. To investigate the chemical and isotopic composition of the local groundwater, three water samples (PB samples) were collected from shallow wells in the surroundings. The water table measured in the sampled wells was less than 2 m below the ground level, suggesting a shallow water circulation typical of aquifers in clayey lithotypes.

Temperature, pH, Electric Conductivity (EC) and Oxidation–Reduction Potential (ORP) were measured directly in the field using portable instruments calibrated against certified materials. Total alkalinity was measured by volumetric titration (HCl 0.1 N) using methyl orange as an indicator. Water samples were filtered through 0.45 µm pore size filters and then stored in HDPE bottles. The aliquot for metal analyses was acidified with HCl to prevent any salt precipitation. Major anions and cations were analyzed by Ion Cromatography (Dionex ICS-3000) and measured concentrations (expressed in mg/L, standard error better than 5%) are referred to NIST traceable standard materials (Thermo-Scientific Certificate). Details of sampling and analytical procedures for dissolved gases (CO_2_, N_2_, O_2_, Ar, He and CH_4_) are reported in ref.^53^. Free gases were collected in pre-evacuated 250 mL flasks, equipped with two teflon stopcocks fixed with a rubber O-ring, using an inverted PE funnel placed over mud volcano gas emissions. The gas composition was then analysed by Gascromatography (Agilent Technologies mod. 6890N) according to the methodology reported in ref.^54^.

## Results

### Geophysical results

#### ERT surveys

**Figure 2:**
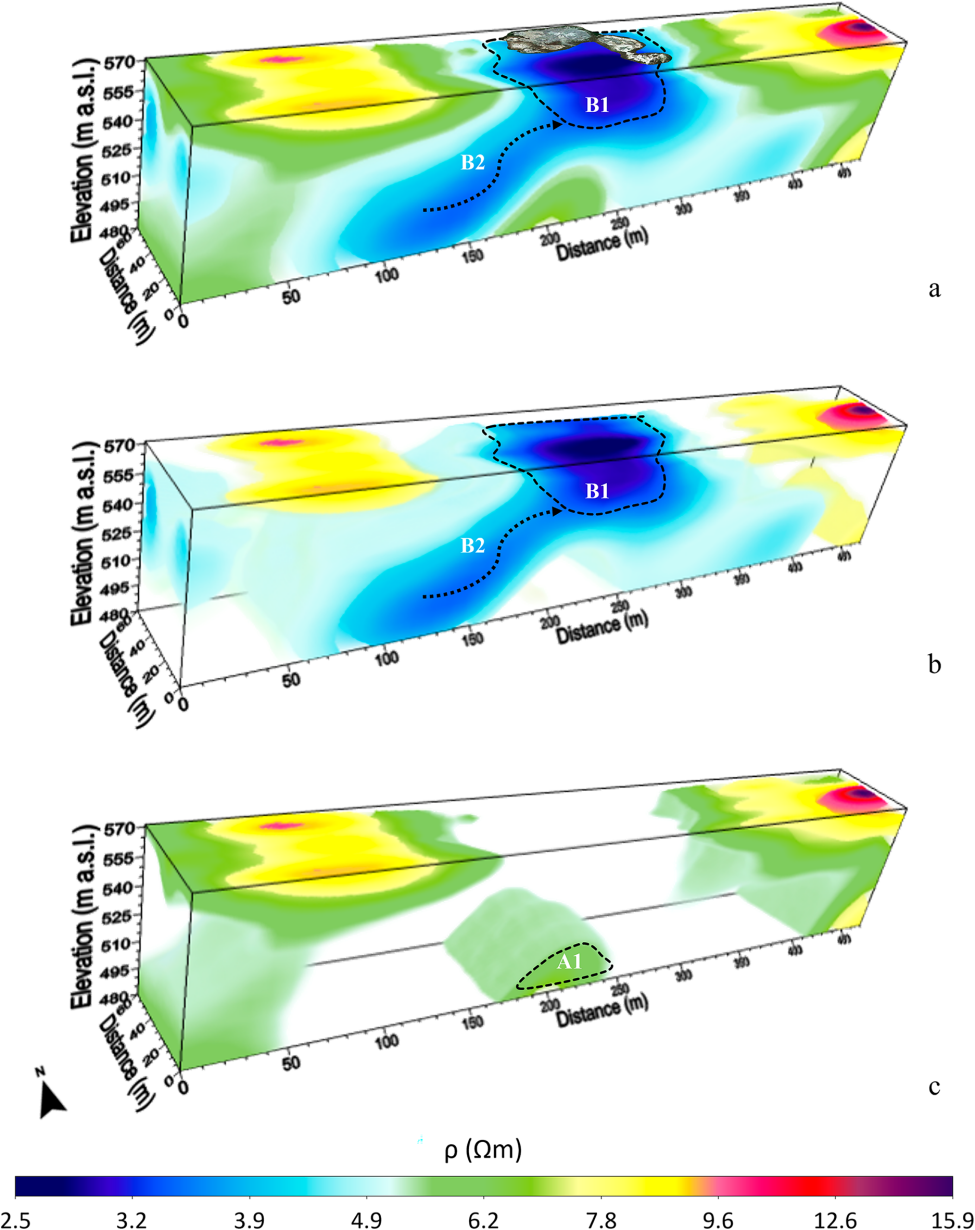
3D inversion results of the 2D ERT data acquired along the four profiles in Fig. 1b in the winter season. Resistivity volumes in the range 2.5 Ωm ≤ ρ ≤ 15.9 Ωm (**a**); 2.5 Ωm ≤ ρ ≤ 4 Ωm, 4.5 Ωm ≤ ρ ≤ 5 Ωm, 7.5 Ωm ≤ ρ ≤ 9 Ωm and 9.5 Ωm ≤ ρ ≤ 15.9 Ωm (**b**); 5.5 Ωm ≤ ρ ≤ 6.5 Ωm, 7.5 Ωm ≤ ρ ≤ 9 Ωm and 9.5 Ωm ≤ ρ ≤ 15.9 Ωm (**c**). The position of the MVs is added on (**a**).

**Figure 3:**
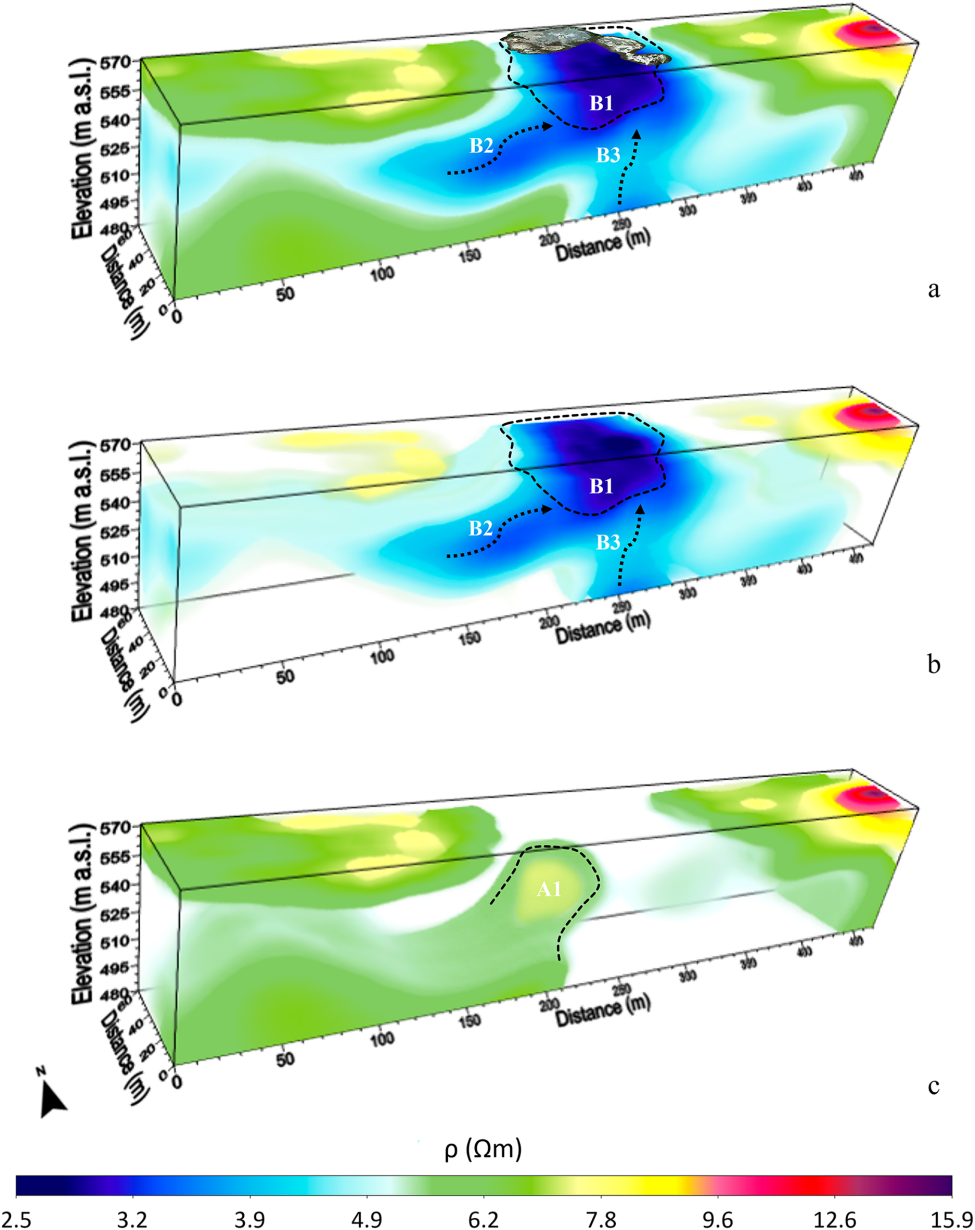
3D inversion results of the 2D ERT data acquired along the four profiles in Fig. 1b in the summer season. Resistivity volumes in the range (**a**) 2.5 Ωm ≤ ρ ≤ 15.9 Ωm; (**b**) 2.5 Ωm ≤ ρ ≤ 4 Ωm, 4.5 Ωm ≤ ρ ≤ 5 Ωm, 7.5 Ωm ≤ ρ ≤ 9 Ωm and 9.5 Ωm ≤ ρ ≤ 15.9 Ωm; (**c**) 5.5 Ωm ≤ ρ ≤ 6.5 Ωm, 7.5 Ωm ≤ ρ ≤ 9 Ωm and 9.5 Ωm ≤ ρ ≤ 15.9 Ωm. The position of the MVs is added on (**a**).

**Figure 4:**
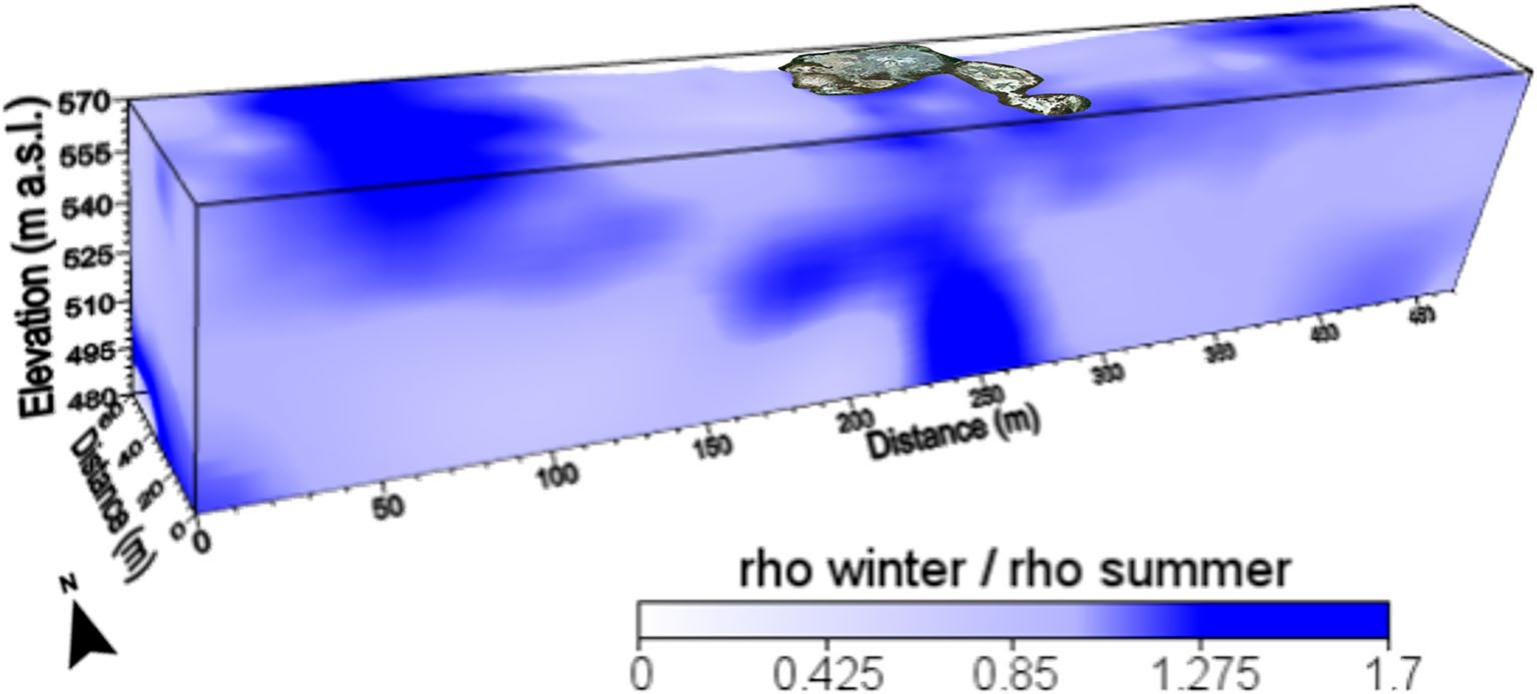
The ratio of the resistivity data observed in the winter (Fig. 2) and summer (Fig. 3) seasons displayed in a 3D plot corresponding to the whole investigated volume.

Figures 2 and 3 show the 3D inversion results of the 2D ERT data acquired during the winter (Fig. 2) and summer (Fig. 3) seasons, which were obtained with an RMS misfit error of 1.99 and 3.36, respectively. The small range of resistivity variations (2.5 Ωm ≤ ρ ≤ 15.9 Ωm) observed in both surveys clearly indicates that the investigated buried volume is essentially formed by a single lithology (clay) under saturated conditions. Therefore, the highlighted weak contrasts in the resistivity values can be attributed to a differential amount of saline water rising along possible fractured zones or fissure networks that provide preferential pathways for fluid migration. The two 3D models in Figs. 2 and 3 highlight a broad relatively high-conductivity anomaly in the central part of the investigated volume, which deepens up to the maximum exploration depth, probably associated with the ascent of muddy fluids. The geometry of this anomaly provides useful clues for defining the paths that CH_4_ is likely to follow to transport saline water to the surface, exploiting areas of greater permeability. In fact, the very low resistivity values that define this anomaly are indicative of a higher saline water input than the rest of the surveyed volume. In particular, the very high conductive B1 anomaly would represent the shallow root of the MVs; instead, the in-depth trends of the other two conductive anomalies (B2 in Fig. 2 and B3 in Fig. 3) seem outline the pathways of the fluids feeding the shallow B1 reservoir. To better identify where the resistivity changes have occurred from winter to summer, which could provide an indication of the fluid flow variation, the ratio of winter to summer resistivity data is shown in Fig. 4. The dark blue zones represent the volumes where the resistivity values in the winter season are greater than those observed in the summer period. Therefore, the dark volumes that characterize the central and eastern parts of the ERT survey area could be associated with a more gas-rich flow permeating the presumed fracture network that would feed the shallow root of the MVs (i.e. anomaly B1 in Figs. 2 and 3). Conversely, the light blue zones, which indicate volumes where the resistivity values decrease slightly or remain essentially the same over time, confirm a high saline water content within the fluid flow migrating from the deep to the shallow subsurface. The distribution of the resistivity ratios in Fig. 4 shows that rising saline water follows well-defined preferential pathways rather than moving uniformly into the shallowest part of the soil.

#### SP surveys

**Figure 5:**
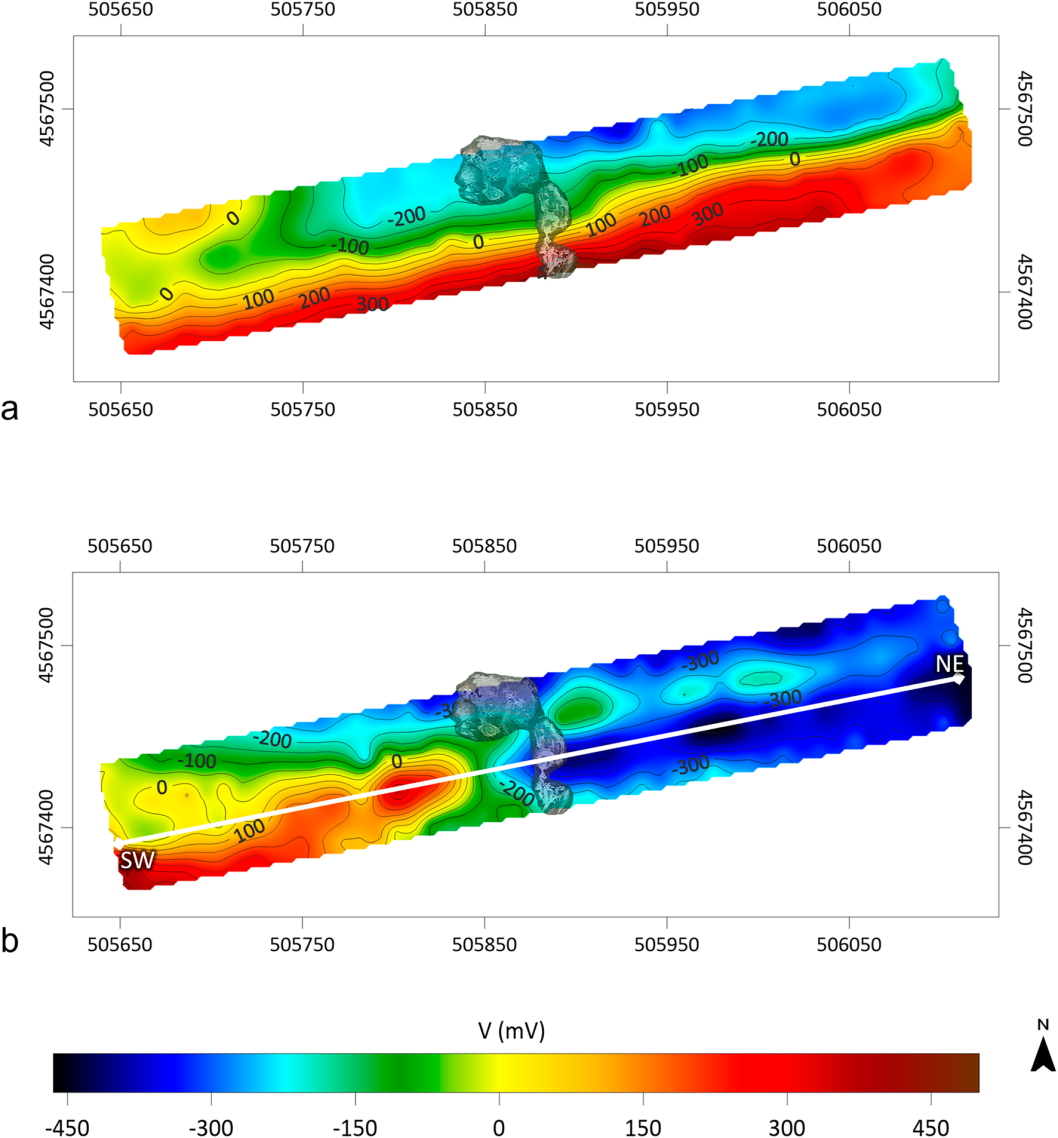
SP anomaly maps obtained from the winter (**a**) and summer (**b**) survey with overlap of MVs position.

**Figure 6:**
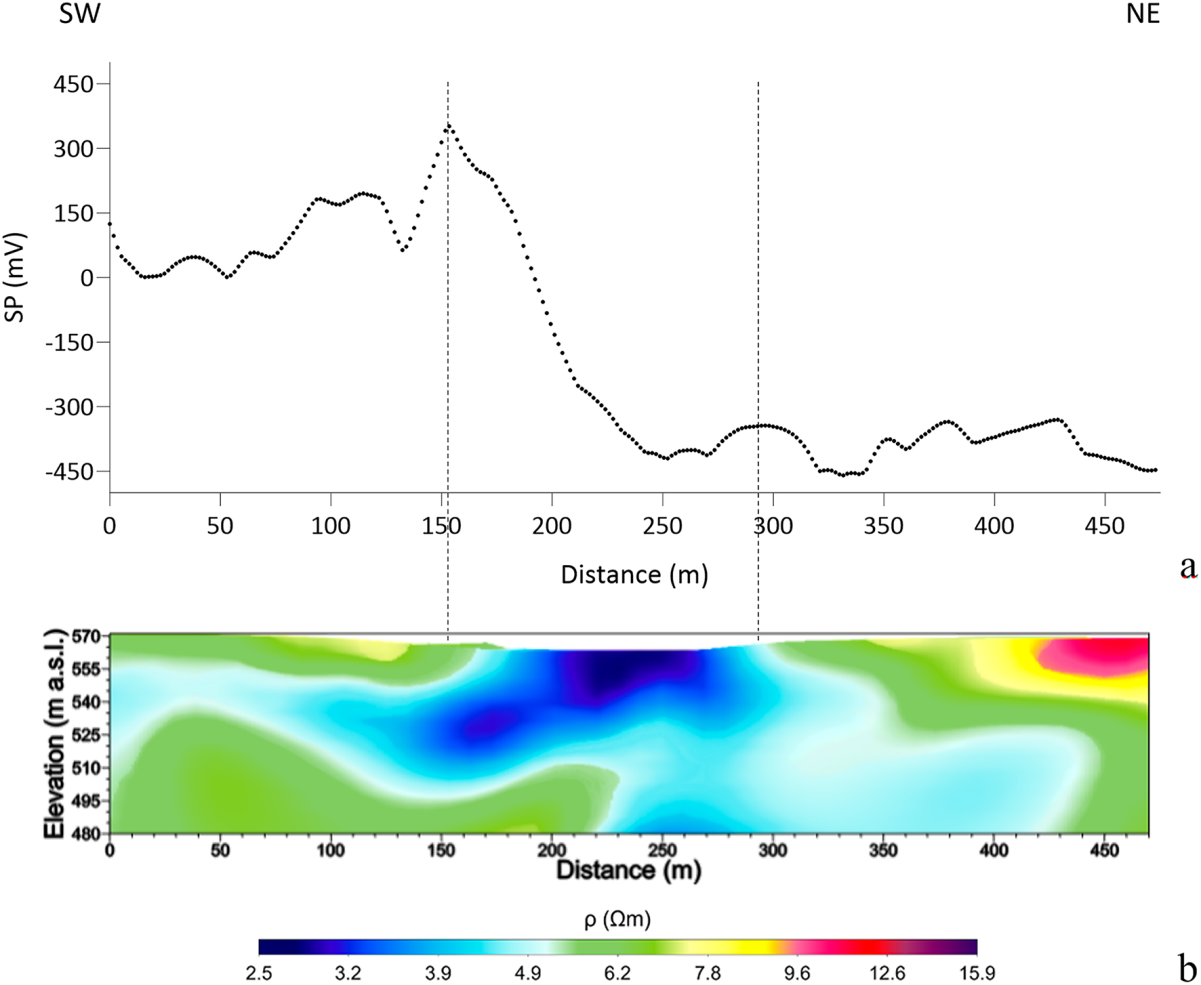
SP anomaly pattern (**a**) and cross-section of the 3D ERT model in Fig. 3 (**b**) along the SW-NE profile in Fig. 5b.

Figure 5 shows the SP maps produced for the winter (Fig. 5a) and summer (Fig. 5b) surveys. For the interpretation of the observed SP anomalies, it is assumed that they are mainly caused by electrokinetic mechanisms induced by fluid flows (i.e. SP primary sources) through fault/fracture systems and/or high permeability zones [Ref.^55^ and references therein]. The winter SP map (Fig. 5a) is dominated by a relatively large-wavelength dipolar electric field oriented approximately SE-NW, which could be generated by a distribution of secondary electric charges induced by primary polarization sources along a deep structural discontinuity with an approximate SW-NE orientation. In contrast, the summer SP map (Fig. 5b) shows positive and negative short-wavelength anomalies superimposed on a longer-wavelength field. In particular, the SP map in Fig. 5b exhibits a general pattern consisting of two domains: one to the SW, characterized by positive SP values, and the other to the NE, where negative values dominate. The geometry of the larger wavelength anomaly separating the two domains suggests the contribution of a relatively deep source responsible for a natural dipolar electric field approximately SW-NE oriented. The latter could therefore indicate the presence of a network of cracks in the clay formation, with an orientation perpendicular to the field direction, along which gases/fluids from deep reservoirs would find a preferential pathway to the surface. The location of this hypothetical fissured zone correlates well both with the position of the MVs visible at the surface (see Fig. 5b) and with the low resistivity pattern in the central part of the investigated volume (Figs. 2 and 3). To better clarify our interpretative hypothesis, Fig. 6 shows the SP anomaly pattern along the SW-NE profile in Fig. 5b overlaid to the corresponding cross-section of the 3D ERT model in Fig. 3. As we can see, the longer wavelength anomaly delimited by the dashed lines in Fig. 6a well matches to the low resistivity area in Fig. 6b. On the other hand, we can tentatively associate the negative short-wavelength SP anomalies observed in the NE portion of the profile (Fig. 6a) with primary electrical charge distributions in the shallower part of the investigated subsurface. These anomalies could be related to biogenic activity, specifically decomposition processes of organic matter that result in oxygen consumption and generation of sub products, such as CO_2_^56^. The high oxygen consumption in turn results in a reducing environment, characterized by SP values that tend to negative^57^. This interpretation would be supported by literature studies^58–61^, which have shown a strong correlation between negative SP anomalies and areas of CO_2_ degassing.

**Figure 7:**
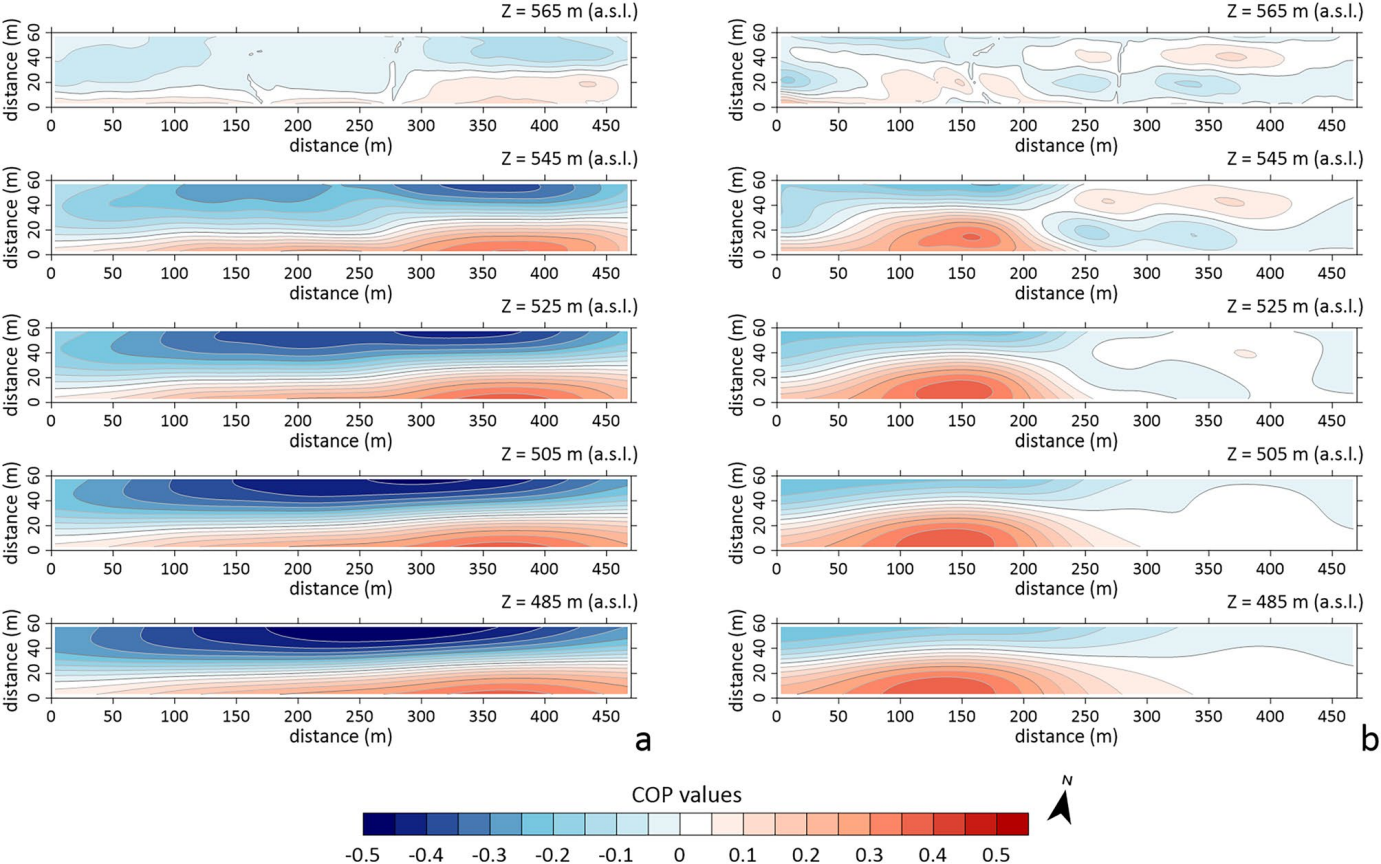
3D tomographic inversion of the SP data collected in the winter (**a**) and summer (**b**) survey.

Figure 7 shows the 3D tomographic inversion results of the SP data collected during the winter (Fig. 5a) and summer (Fig. 5b) surveys. The tomography is represented by slices in the depth range between 565 and 485 m a.s.l., which allowed the highest probability central nuclei to be defined with good resolution^38^. As it can be seen in Fig. 7a, there is a clear bipolar trend, approximately SE-NW, for which the depth of its source (i.e. maximum positive and negative charge accumulations) cannot be identified due to both the size of the survey area and the spatial sampling (10 m) of the SP data. In fact, the survey parameters do not allow adequate resolution of natural electric fields likely generated by secondary charge distributions along structural discontinuities permeable to deep fluid flows. In contrast, Fig. 7b shows the highest values of positive and negative charge nuclei, probably responsible of the relatively long-wavelength SP anomaly in Figs. 5b and 6a, concentrated at depths between 560 and 540 m a.s.l. The approximately SW-NE orientation of this bipolar distribution could be related to the SE-NW oriented network of fissures suggested by the qualitative analysis of the SP map in Fig. 5b.

### Geochemical results

#### Soil diffuse emissions

A total of 171 soil gas flux measurements were carried out throughout the investigated area (13.7 ha). Statistical summary of the investigated variables is given in Table 2.

**Table 2:** Statistical summary of chemical and isotopic variables.

	Mean	Max	Min	SD	25th percentile	75th percentile
CO_2_ flux (g/m^2^day)	9.6	47.7	0.0	7.0	5.9	11.2
CH_4_ flux (mg/m^2^day)*	46.1	247.1	2.5	59.7	7.7	79.0
T (°C)	10.2	12.6	7.1	1.2	9.4	11.1
^13^C efflux (‰)**	− 22.7	− 9.1	− 37.9	6.7	− 27.8	− 17.6

*SD* standard deviation.

*CH_4_ flux row referred to 43 measured points, **^13^C efflux is referred to 55 measured points.

**Figure 8:**
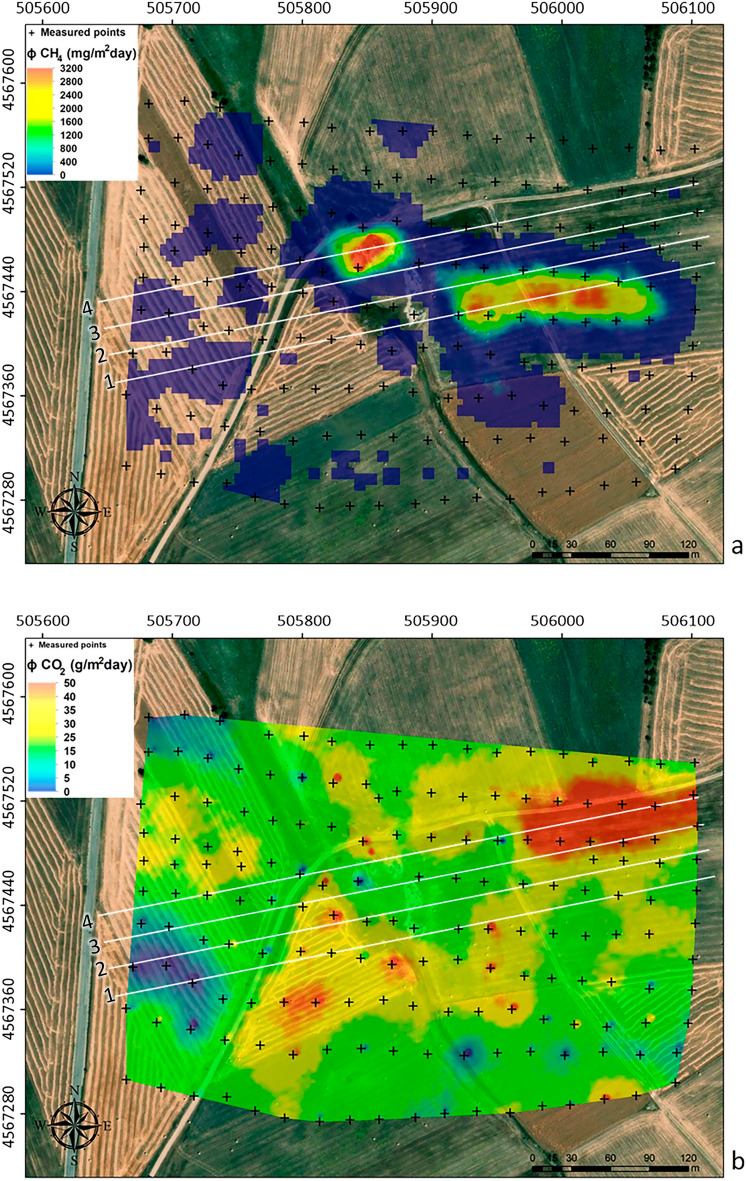
(**a**) CH_4_ and (**b**) CO_2_ map built through sGs algorithm with the four ERT profiles (white lines) overlaid. The blue/green and the anomalous yellow/red areas in (**a**) are representative of background and endogenous population, respectively. The CO_2_ fluxes shown in (**b**) are typical of the soil background values. Therefore, the different colors can only be attributed to the different types of vegetation cover. The figure was created using the WinGslib 1.5.6 open-source software (http://www.gslib.com/) and postprocessed through the ArcMap 10.2.2 software (https://www.esri.com/en-us/arcgis/products/arcgis-desktop/resources).

**Figure 9:**
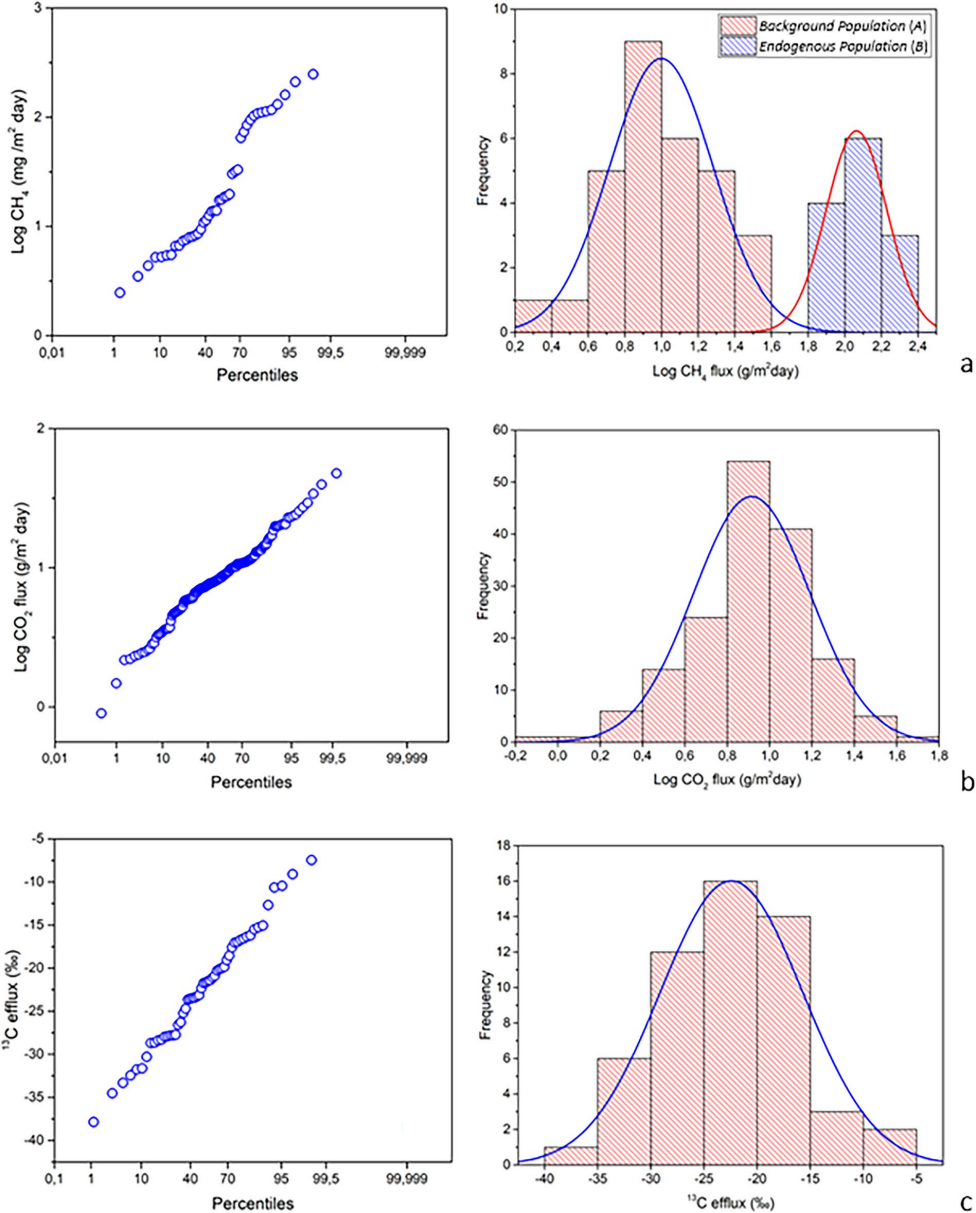
(**a**) CH_4_ PP-plot and histograms; (**b**) CO_2_ PP-plot and histograms; (**c**) ^13^C efflux PP-plot.

Soil CH_4_ flux ranges from 0 to ~ 3000 mg/m^2^ day with an average value of 11.74 mg/m^2^day. Only at 43 measurement points a soil CH_4_ flux greater than 0 mg/m^2^day was detected. As shown in Fig. 8a, the highest values of methane flux were measured in the area where the mud volcanoes are located. Moreover, this higher flux anomaly extends eastwards into a morphologically elevated area, while lower values of CH_4_ fluxes were detected in the other peripheral sectors. The occurrence of two CH_4_ flux statistical populations is inferred from the logarithmic cumulative probability plot in Fig. 9a, where the presence of an inflection point in the data distribution is evident. The graphical statistical approach (GSA)^40^ was used to partition this distribution into two distinct lognormal populations: Population *A*, which represents the 68.4% of the total statistical sample and is representative of the marginal areas (Fig. 9a), and Population *B* (mud volcanoes area), which represents the remaining 31.6% of the statistical samples. The total output estimation returns a value of about 7.5 kg/day, which is a non-negligible value and comparable to that measured in persistent degassing volcanoes^62^.

**Figure 10:**
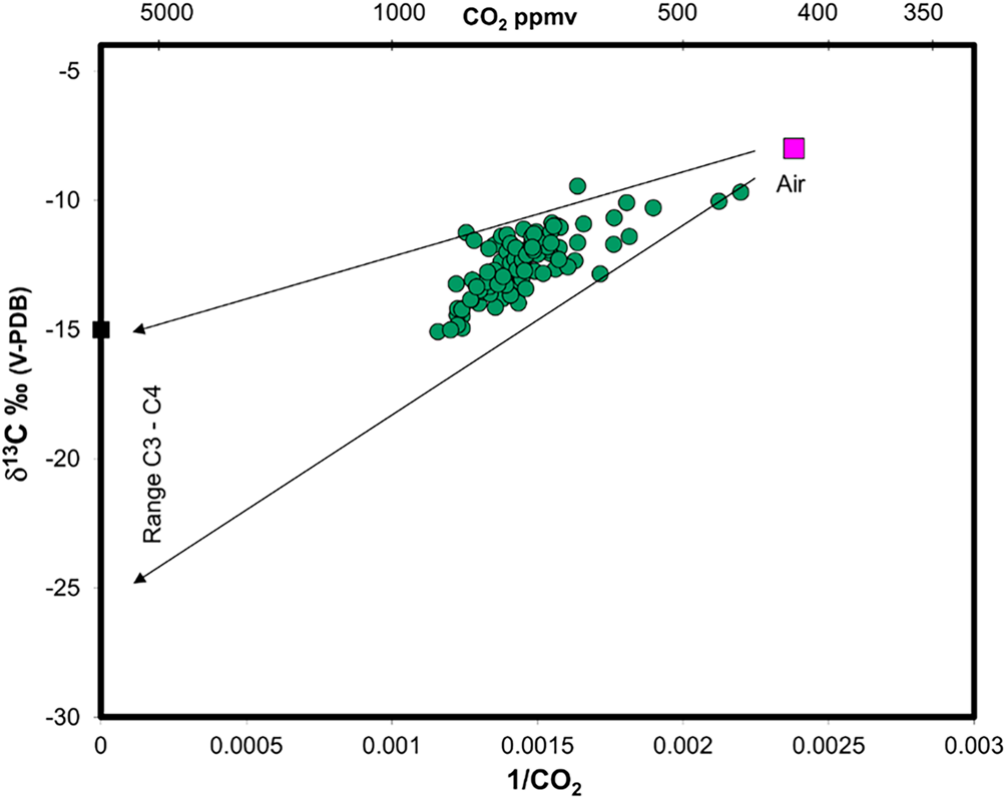
δ^13^C efflux vs CO_2_ concentration scatterplot.

Soil CO_2_ fluxes (Fig. 8b), measured contemporaneously with methane measurements, range from 0 to 47.7 g/m^2^day with an average value of 9.63 g/m^2^day, which is the typical range of soil background values. The absence of inflection points and the single modal distribution highlighted in Fig. 9b prove the existence of a single statistical population of CO_2_ fluxes with values typical of biological background. This result is corroborated by the isotopic data; in fact, as for the δ^13^C efflux (estimated at 56 points, see Sect. “Geochemical measurements”), it varies in a range from − 37.86‰ to − 9.1‰ with an average value of − 22.7‰, which is the typical range of biologically derived gas (Fig. 9c). As shown in Fig. 10, all samples are in the typical range of C3-C4 plants, strongly supporting a purely biological origin for the CO_2_ degassed from the soil. Therefore, the differences highlighted in the CO_2_ map (Fig. 8b) are exclusively due to the different types of vegetation cover of the investigated area during the geochemical survey (barren soil vs. vegetation-covered soil). Finally, the soil temperature ranges from 7.1 to 12.6 °C with an average value of 10.2 °C.

#### Sampled fluids geochemistry

**Figure 11:**
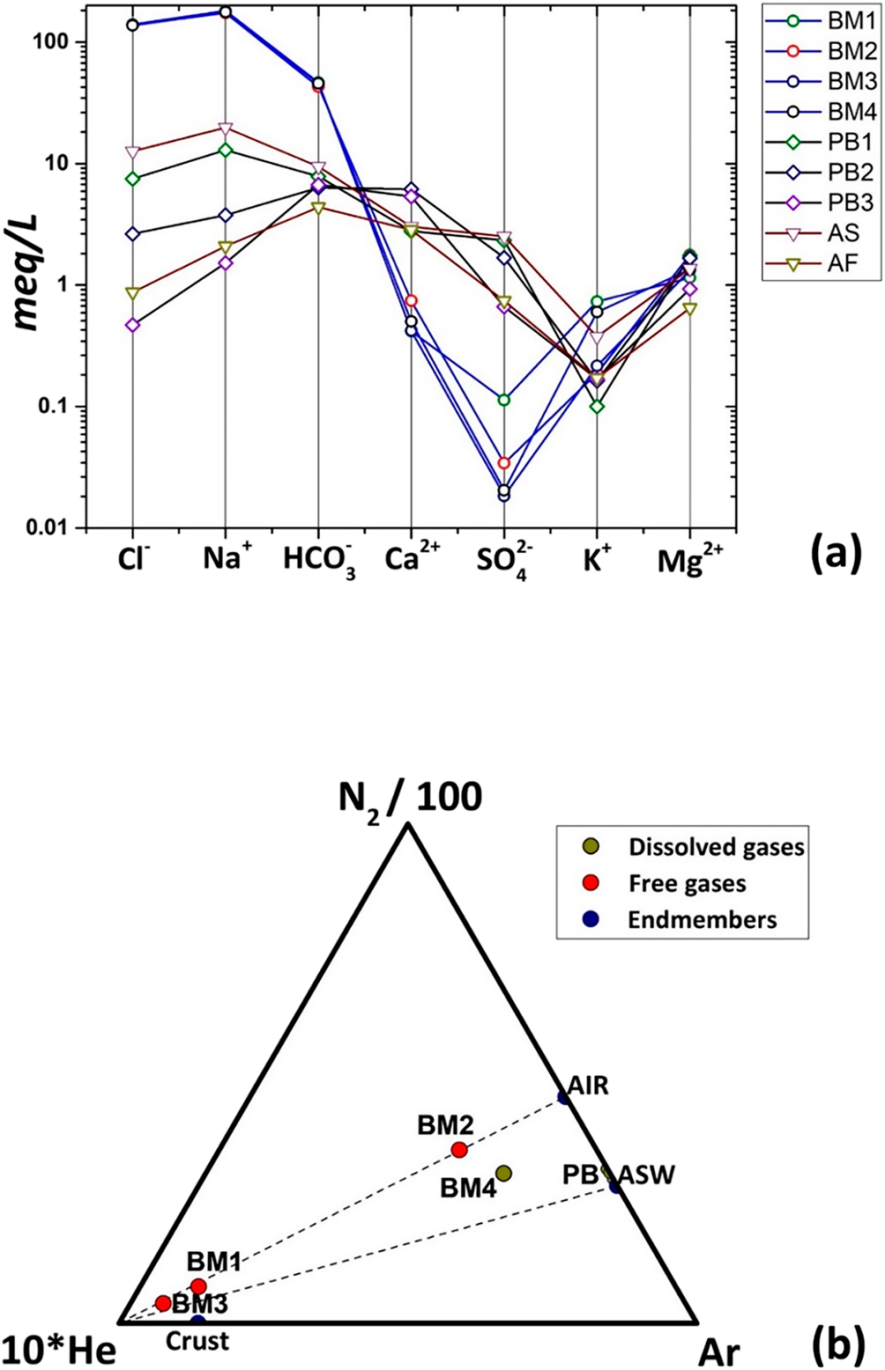
(**a**) Shoeller diagram of whole chemical composition of sampled waters. (**b**) Ternary plot of unreactive gases detected in the fluid samples. The endmembers for AIR (N_2_/Ar = 83), Air Saturated Waters (ASW, N_2_/Ar = 38), and Crust are also reported on the plot. The three PB samples are coincident in the plot.

The results of the chemical analyses are given in Table 1. The main chemical composition is shown in Fig. 11a using the Shoeller diagram^63^. The waters discharged from the mud volcanoes (BM samples) are of Na-Cl, HCO_3_ type, while the samples from local wells and runoff water (PB2, PB3 and AF) are of Ca, Na-HCO_3_ type. The difference in water type between BM and the shallow hydrogeological system (PB, AF and AS samples) is also evident from the remarkable difference in salinity (18 mS/cm vs. less than 2 mS/cm, Table 1). The PB2 water solution shows additional SO_4_ attributed to agricultural soil leaching, as also suggested by the highest NO_3_ concentration detected in this well (48 mg/L vs. less than 10 mg/L in other sampled waters)^64^. The overall chemical composition and salinity of the BM water suggest a different origin as compared to the shallow hydrogeological system. As a result, the BM water appears to rise at mud volcanoes without mixing with the shallow hydrogeological system.

The BM water is also characterized by relatively large amount of Br (27 mg/L), F (9–12 mg/L) and HCO_3_ (2.8 g/L). These results support the dynamics of BM waters rising from a relatively deep reservoir or, better, water trapped in sediments highly mineralized by prolonged interaction with the host terrigenous rock and endogenous gases^16^. The proposed hydrodynamics is associated with weak but continuous gas bubbling; therefore, it seems realistic to attribute the water spring to the gas discharge. The presence of endogenous gas reservoirs has been identified in the basement of this sector of the southern Apennines^65^. In order to investigate the source of these fluids (Table 1), the ternary diagram of the unreactive gas species (N_2_, He, Ar)^66^ has been reported in Fig. 11b. As expected, the shallow hydrogeological system (PB samples) is saturated only by atmospheric component; in fact, the relative Ar-N_2_-He compositions plot close to the Air Saturated Water (ASW) endmember, as it is also clearly defined by the N_2_ and O_2_ predominance in these samples (see Table 1). Differently dissolved gases compositions of the BM samples have an endogenous source identified with a longer residence time in the crustal geological system, and also confirmed by the remarkable He content (≈ 500 ppm) generated by radioactive decay of lithophile elements Uranium and Thorium in the host rocks^67^. Among the BM samples plotted, BM2 and BM4 are shown to have undergone air contamination during the sampling, whereas BM3 sample seems to be the more representative of the fluids sampled from depth, as it also pointed out by the most reductant water ORP value (− 177 mV, Table 1). The chemical composition of the free BM gases discharged from the mud volcanoes (Table 1) shows that the gases are manly composed of CH_4_ (≈ 95%) with a minor amount of CO_2_ of about 1% in agreement with previous compositions reported by ref.^68^ and references therein.

Summing up, the Bolle della Malvizza mud volcanoes are the surface manifestation of the presence of an ascending fluids plume from a deep pressurized CH_4_ gas reservoir of thermogenic origin^68^. The upraising fluids plume is composed of CH_4_ and highly saline waters, which can be associated with the same CH_4_ source or transported to the surface by the rising gas, acting as a gas carrier.

## Discussion

The criteria for identifying suitable underground environments for gas storage are mainly geological. Indeed, a potential storage site must have adequate structural stability, volumetry, porosity, permeability, and appropriate isolation of the permeating fluids from the atmosphere as well as from surface and/or groundwater bodies (i.e. lakes, rivers, seas, exploited aquifers). As a guide, a site is generally considered suitable for storage if: (i) it is sufficiently deep (more than 800 m below the surface), (ii) the thickness of the rock that can contain the gas (reservoir) is significant (in the order of 1000 m or more), and (iii) considerable thicknesses (hundreds of meters) of impermeable rock (caprock) are available above the surface to prevent carbon dioxide stored at depth from rising to the surface because of its high mobility and low density. This third condition means that geological settings incapable of containing fluids cannot be considered for carbon dioxide storage. Such unfavorable conditions are generally found in natural systems characterized by the presence of faults and/or fracture systems coupled or not with the presence of more permeable geological layers that act as preferential pathways for fluids rising to the surface.

**Figure 12:**
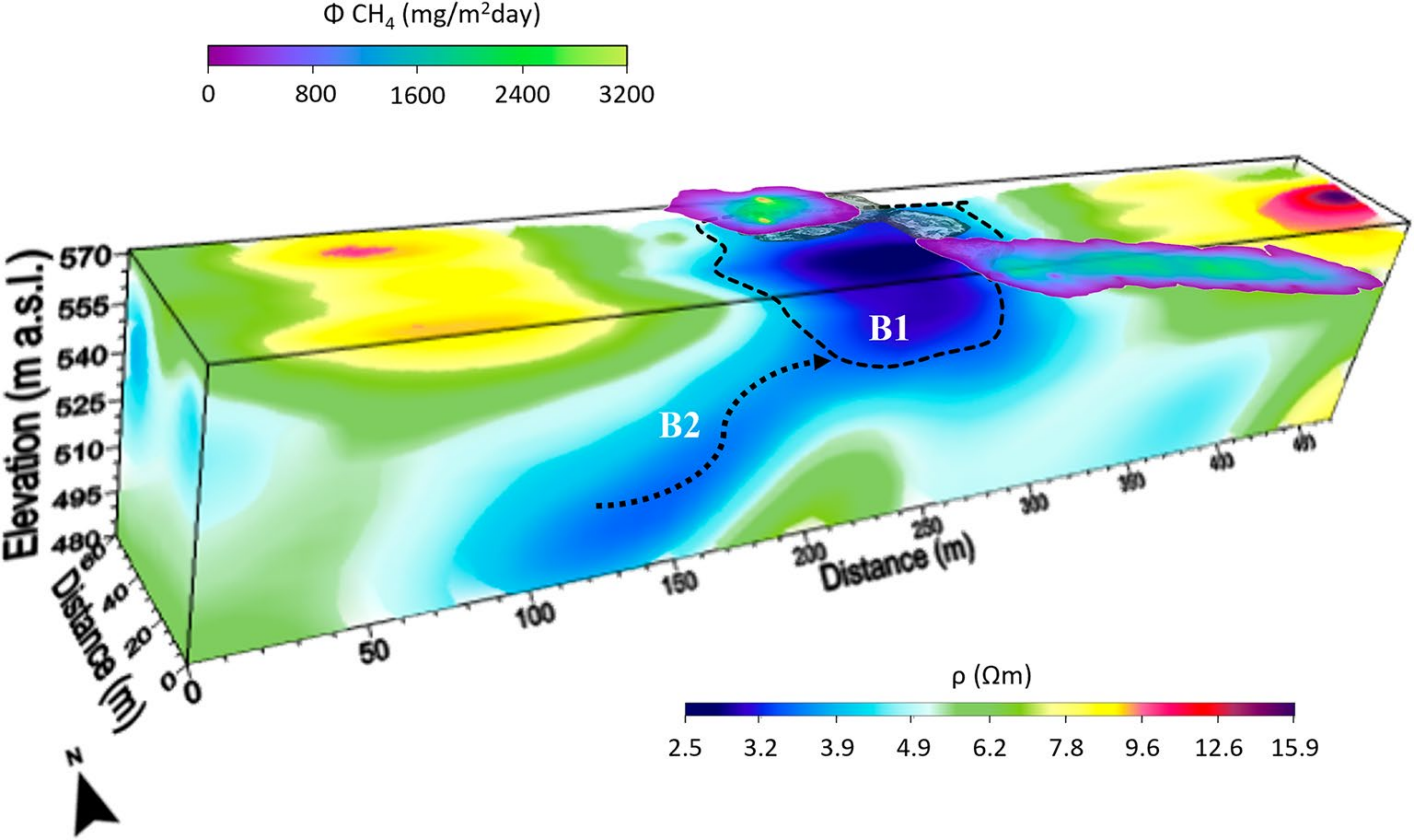
The higher CH_4_ flux anomalies (see Fig. 8a) are superimposed on the 3D resistivity model in Fig. 2a.

The Bolle della Malvizza natural gas reservoir was considered to test the feasibility of a combined use of high-resolution geoelectrical investigations to define geological-structural features and processes controlling potential gas leaks. The ultimate goal of our research is to propose methodologies for monitoring gas or, more generally, fluids leakage in storage reservoirs, which may also be applicable in the context of geological CO_2_ sequestration. The study area is tectonically active and is characterized by ascending dynamics of endogenous fluids, which testifies to the occurrence of possible discontinuities among impermeable layers of terrigenous formations. These discontinuities may result from the presence of more porous sedimentary formations (such as sands or heterogeneous sand-rich layers), intercalations and/or tectonic discontinuities, i.e. faults and fractures, which can increase the permeability and thus allow the migration of fluids under pressure gradients. In fact, the Bolle della Malvizza mud volcanoes are the surface manifestation of the rising of deep fluids, mainly composed of methane gas, which likely strips saline water trapped into sediments. This process is strongly localized in the mud volcanoes area, as confirmed by diffuse emission measurements (Fig. 8) and water characteristics of the studied area (Table 1). The fluids that rise before reaching the surface do not appear to mix with any lateral hydrogeological system and therefore are localized in a unique vertical conduit. This would be confirmed by the pattern of the large conductive anomaly observed in the central sector of the ERT survey area (Figs. 2 and 3). In fact, the very low resistivity values recorded up to the maximum exploration depth would be justified by the highly saline waters transported to the surface by rising gas (Sect. "Sampled fluids geochemistry"). However, overlaying the soil diffuse CH_4_ flux observed during the winter season on the 3D resistivity model in Fig. 2a (see Fig. 12), the highest methane gas fluxes are observed mainly in the north-central and eastern parts of the ERT survey area. In particular, the most conductive areas overlap the water findings above the mud volcanoes. In practice, the most conductive zones likely represent the upwelling conduits of endogenous fluids. The ascent of saline waters could be driven by a gas phase, composed mainly of methane, formed at depth. As methane is poorly soluble in water, it could separate from the liquid as the fluid rises and the pressure decreases, forming a gas phase that rises to the surface mainly coupled with the saline liquids in the conduits that form the MVs. However, the gas phase can also decouple from the saline waters by rising through discontinuities that allow it to reach the surface in other areas. This possibility could partly explain the relatively high CH_4_ flux values measured on the hill in the eastern part of the investigated area, where a possible pathway for gas ascent is suggested by comparing winter and summer resistivity measurements (Sect. "ERT surveys" and Fig. 4).

The application of the proposed approach has demonstrated its potential both for detailed characterization of the impermeable rock formation and for monitoring potential fluids flow through areas of greater permeability. In particular, the resistivity model retrieved for the investigated subsurface volume agrees well with a geological environment formed by clays and flaky clays with levels of marly and arenaceous limestone, as inferred from recent geological-structural studies^24,25^. The weak resistivity contrasts are consistent with physical discontinuities attributable to variations in porosity, permeability and fracturing of the formation under investigation. These discontinuities appear to be controlled by the presence of two structural lineaments, oriented SW-NE and SE-NW, respectively (Sect. "SP surveys"), previously suggested by tectonostratigraphic analyses^25^. Moreover, the identified orientations correspond well to the two alignments of the mud volcanoes visible on the surface and could therefore indicate the presence of buried faults along which gases (and liquids) from deep sources would find a preferential pathway to the surface (see Fig. 1). This assumption also seems to be confirmed by the variation in the resistivity anomaly patterns emphasized in Fig. 4, which highlights the preferential upward pathways that waters and gases follow as they rise to the surface.

In turn, the SP prospecting has provided support for the interpretative hypotheses suggested by the ERT data modeling, which, due to the very narrow range of the observed resistivity variations, necessarily require validation by additional data and/or information available for the survey area. In particular, the orientation of the relatively large-wavelength dipolar electric field visible on the winter SP map (Fig. 5a) seems to indicate the presence of a deep SW-NE oriented discontinuity, which cannot be identified by our survey due to the small size of the investigated area and the short interelectrode distance, as also confirmed by the SP inversion results (Fig. 7a). On the other hand, the longer-wavelength SP anomaly observed on the summer SP map (Fig. 5b) supports the occurrence of a shallow fractured zone in the clay formation as inferred from the 3D ERT model in Fig. 3, which identifies a low resistivity pattern in the central sector of the investigated volume visible up to the maximum exploration depth. Furthermore, the inversion of the SP dataset localizes the maximum/minimum COP values in the depth range from 560 to 540 m a.s.l., which is consistent with the root of the MVs, i.e. the B1 anomaly in Figs. 2 and 3.

## Conclusions

A multi-methodological geophysical approach, based on high-resolution ERT and SP prospections, has been proposed as a tool for identification and monitoring of possible migration pathways of fluid/gas to the surface from geological gas storage sites. Its effectiveness has been evaluated by carrying out 3D high-resolution ERT and SP surveys over time in a test area featured by natural gas emissions, whose geological setting could well resemble a geological formation for anthropogenic carbon dioxide storage. As main findings, we note that the ERT prospecting (Figs. 2 and 3) has shown a high sensitivity of the resistivity parameter even to small variations in the permeability of the assumed caprock, probably due to the presence of a network of cracks/fractures, as well as its ability to detect changes in the geometry of the resistivity anomalies over time, which may be indicative of upwelling pathways of fluids in the gas phase and/or dissolved in water. For its part, the SP monitoring proved useful in identifying fluids migration towards the surface thanks to the detection of electrical charge accumulations (Fig. 7), which are likely to be responsible for increased fluid/gas inflow along the hypothesized fracture system. It is worth noting that the interpretative hypotheses provided by the proposed geophysical approach are overall in agreement with the results of the geochemical investigations performed in the test area, thus confirming its capability to detect and monitor potential fluid/gas flows in the caprock, which are likely to migrate from geological storage reservoirs to the surface along permeable fracture systems.

In this regard, it should be emphasized that the integration of high-resolution geophysical models with geological and geochemical data allows a very detailed reconstruction of the architecture of gas storage systems, which is fundamental for numerical simulations of gas leakage from a geological reservoir to study its evolution over time.

## Data Availability

The datasets used and/or analyzed during the current study are available from the corresponding author on reasonable request.
